# EEG features in young female depressive patients with ultra-high risk of psychosis

**DOI:** 10.1192/j.eurpsy.2025.1333

**Published:** 2025-08-26

**Authors:** A. F. Iznak, ?. V. Iznak, E. V. Damyanovich, I. V. Oleichik

**Affiliations:** 1 Laboratory of Neurophysiology; 2 Department of Endogenous Mental Disorders and Affective States, Mental Health Research Centre, Moscow, Russian Federation

## Abstract

**Introduction:**

One of the directions of scientific research in modern psychiatry is the search for predictors of the developing of a manifest psychotic attack in patients with non-psychotic disorders, that is, predictors of an ultra-high risk of psychosis. Such predictors may include various biomarkers, for example, the quantitative EEG parameters, which subtly reflect the features of the brain functional state.

**Objectives:**

The aim of the study was to search for EEG correlates of the brain functional state in depressive patients of the group of an ultra-high risk of developing psychosis in comparison with EEG parameters of patients without symptoms of a risk of psychosis.

**Methods:**

The study included 74 female depressive patients (F31.3-4, F32.1-2, by ICD-10) aged 16-26 years (mean age 20,8±3,5 years) at the stage of remission establishing after treatment course, clinically divided into two groups. First group consisted of 32 patients with depressive conditions and attenuated prodromal psychotic symptoms assessed by SANS and SAPS scales and by COGDIS and COPER criteria, but without a history of manifest psychotic attack. The second group included 42 depressive patients without symptoms of ultra-high risk of developing psychosis. In all patients, multichannel background EEG was recorded with spectral power analysis in narrow frequency sub-bands. Intergroup EEG differences were statistically analized using Mann-Whitney test.

**Results:**

According to EEG data, the functional state of the cerebral cortex in patients of group 2 at the stage of remission establishing was approaching normal. The EEG of the 1st group of patients differed from the EEG of the 2nd group by significantly lower values of EEG spectral power in the alpha3 sub-band (11-13 Hz) in the occipital leads and by significantly increased spectral power of theta1 (4-6 Hz) activity in the central-parietal areas. Such EEG frequency structure of patients in group 1 reflects a reduced functional state of associative areas, and may also indicate dysfunction of the frontal parts of the cerebral cortex. These EEG features of patients in group 1 are consistent with a significantly greater severity of their positive and negative symptoms scores by the SAPS and SANS scales compared to group 2.

**Conclusions:**

In depressive patients at the stage of remission establishing who have symptoms of an ultra-high risk of developing psychosis a reduced functional state of the associative and frontal areas of the cerebral cortex is noted, which may underlie the characteristics of their clinical conditions.

**Disclosure of Interest:**

None Declared

